# The HER2 inhibitor TAK165 Sensitizes Human Acute Myeloid Leukemia Cells to Retinoic Acid-Induced Myeloid Differentiation by activating MEK/ERK mediated RARα/STAT1 axis

**DOI:** 10.1038/srep24589

**Published:** 2016-04-14

**Authors:** Xuejing Shao, Yujia Liu, Yangling Li, Miao Xian, Qian Zhou, Bo Yang, Meidan Ying, Qiaojun He

**Affiliations:** 1 Zhejiang Province Key Laboratory of Anti-Cancer Drug Research, Institute of Pharmacology and Toxicology, School of Pharmaceutical Sciences, Zhejiang University, Hangzhou, Zhejiang, China

## Abstract

The success of all-trans retinoic acid (ATRA) in differentiation therapy for patients with acute promyelocytic leukemia (APL) highly encourages researches to apply this therapy to other types of acute myeloid leukemia (AML). However, AML, with the exception of APL, fails to respond to differentiation therapy. Therefore, research strategies to further sensitize cells to retinoids and to extend the range of AMLs that respond to retinoids beyond APLs are urgently needed. In this study, we showed that TAK165, a HER2 inhibitor, exhibited a strong synergy with ATRA to promote AML cell differentiation. We observed that TAK165 sensitized the AML cells to ATRA-induced cell growth inhibition, G0/G1 phase arrest, CD11b expression, mature morphologic changes, NBT reduction and myeloid regulator expression. Unexpectedly, HER2 pathway might not be essential for TAK165-enhanced differentiation when combined with ATRA, while the enhanced differentiation was dependent on the activation of the RARα/STAT1 axis. Furthermore, the MEK/ERK cascade regulated the activation of STAT1. Taken together, our study is the first to evaluate the synergy of TAK165 and ATRA in AML cell differentiation and to assess new opportunities for the combination of TAK165 and ATRA as a promising approach for future differentiation therapy.

Because all-trans retinoic acid (ATRA; Fig. 1a) was successfully employed for the treatment of acute promyelocytic leukemias (APLs), which are a distinct subtype of acute myeloid leukemia (AML), it has opened new perspectives for differentiation therapy^1^,^2^. However, the use of ATRA as a single agent is not approved for the clinical management of leukemia with the exception of APLs. Therefore, a new differentiation therapy that improves the effectiveness of ATRA and extends the range of myeloid malignancies that respond to retinoids beyond APLs is urgently needed. One possible means for overcoming these problems might be the use of a combination of ATRA with other agents.

Human epidermal growth factor receptor 2 (HER2; erbB2) is a member of the ErbB family, which plays a fundamental role in the regulation of mammalian cell survival, proliferation, adhesion, and differentiation^3^,^4^,^5^. Several studies demonstrate that the inhibition of the HER2 pathway may be a potential therapeutic for leukemia. HER2 was amplified within a Myelodysplastic Syndrome (MDS) patient who developed AML^6^ and Herceptin, which targets the HER2 cell-surface receptor, also showed efficacy in refractory/relapsed HER2-positive adult B-acute lymphoblastic leukemia (B-ALL) patients^7^,^8^. Mubritinib (TAK165; Fig. 1a) is a selective inhibitor of HER2 that is under development by Takeda for the treatment of cancer. Studies show that TAK165 exhibits an antitumor effect on a variety of human cancer cells, including AMLs, by inducing apoptosis^9^,^10^,^11^. However, TAK165 has rarely been reported to regulate the ATRA-mediated differentiation of AML cells.

In the present study, we observed significant synergy between TAK165 and ATRA when they were used in combination against human AML cells. We demonstrate that the enhanced differentiation might be associated with the RARα/STAT1 axis activation rather than HER2 inhibition. STAT1 knockdown significantly decreased the differentiating effect of TAK165 and ATRA. Moreover, we found that the TAK165- and ATRA- induced STAT1 activation was MEK/ERK dependent. Collectively, this study evaluated the capacity of TAK165 to synergize with ATRA in AML cells and induce differentiation, and thus, suggests that this combination therapy is a promising approach as a future differentiation therapy.

## Materials and Methods

### Cells and reagents

Human myeloid leukemia HL60 cells and human breast cancer BT474 cells were purchased from the Shanghai Institute of Biochemistry and Cell Biology (Shanghai, China). Human myeloid leukemia NB4 cells and the HL60 resistant cell line HL60R were gifts from Dr. Lingtao Wu (University of Southern California, Los Angeles). Upon arrival in our laboratory, the cells were grown and were frozen as seed stocks as they became available. Both cell lines were passaged for a maximum of 2 months, after which, new seed stocks were thawed. Both of the cell lines were authenticated using DNA fingerprinting (variable number of tandem repeats), confirming that no cross-contamination occurred during this study. Both of the cell lines were tested for mycoplasma contamination at least every month. The HL60, HL60R and NB4 cell lines were cultured in RPMI-1640 media (Gibco BRL). The 293FT cells were cultured in Dulbecco’s Modified Eagle Medium. All of the media were supplemented with 10% fetal calf serum (Gibco BRL) and 1% penicillin/streptomycin. The cell lines were maintained at 37 °C in a humidified atmosphere containing 5% CO_2_. Primary cells from AML patients (Children’s Hospital of Zhejiang University School of Medicine) were isolated using lymphocyte monocyte separation medium.

ATRA was purchased from Sigma and dissolved in ethanol. Mubritinib (TAK165) was purchased from Selleck. Nitrobluetetrazolium (NBT), PD98059, U0126, and sp600125 were from Calbiochem (San Diego, CA). SB203580 was obtained from MERCK. They all were dissolved in DMSO and stored at −20 °C. In all of the experiments, the final DMSO solvent concentration was ≤0.2% (v/v).

### Cellular proliferation, Cell Cycle Analysis

The total cell number and the viability were assessed by trypan blue exclusion with manual counting in Burkerchambers. The cell cycle distribution was detected by a flow cytometry measurement of the DNA content after the cells were incubated with RNase A (10 μg/ml) and propidium iodide (50 μg/ml). The cellular DNA content was analyzed on a FACS Calibur flow cytometer using the Cell Quest Pro software (BD Biosciences, San Jose, CA). A percentage of each population was measured using Mod FIT software (BD Biosciences). At least 20 000 cells were analyzed for each data point.

### Differentiation detection

The induction of cell differentiation was determined by assessing morphologic changes, CD11b expression, and a nitro blue tetrazolium (NBT) reduction assay.

To assess CD11b expression, the cells (1 × 10^6^) were harvested and washed with PBS, blocked with 3% bovine serum albumin (BSA) in PBS for 30 minutes, and incubated with anti-human CD11b antibody (PE conjugated) for 45 minutes on ice. After incubation, the CD11b expression levels were analyzed with a FACS Calibur flow cytometer (BD Biosciences).

To assess NBT reduction, the cells (5 × 10^5^) were harvested and incubated with PBS containing NBT (1 mg/mL) and freshly diluted 12-O-tetradecanoylphorbol-l3-acetate (TPA; 1 mg/mL) at 37 °C for 30 minutes. The cytospin slides were prepared and examined for cells containing precipitated formazan particles. At least 200 cells were assessed for each experiment.

The cell morphology was evaluated by Wright-Giemsa staining. The cytospin preparations were fixed with methanol and air-dried. The slides were then stained with the Wright–Giemsa solution and examined with a Leica microscope, and the images were captured with a Leica DFC300 FX charge coupled device camera.

### Western blotting

Protein extracts were resolved by 8% to 15% SDS-PAGE. The proteins were transferred onto polyvinylidenedifluoride (PVDF) membranes; the membranes were blocked with 5% nonfat dry milk and incubated with primary antibodies. Antibodies against c-Myc (3G32), p-STAT1 (Tyr701), ERK1 (K-23), JNK (FL), MEK1/2, p38 (H-147), p-MEK-1 (Thr291), p-ERK1/2 (Thr204), and GAPDH (FL-335) were obtained from Santa Cruz Biotechnology (Santa Cruz, CA). PU.1, p21 waf1/CIP1, p-STAT1 (Ser727), p-p38, MAPK (Thr180/Tyr182), p27/Kip1 (D37H1), STAT1, C/EBPβ (LAP), and p-SAPK/JNK (Thr183/Tyr185) antibodies were purchased from Cell Signaling Technology (Danvers, MA). The western blot was visualized using HRP-conjugated secondary antibodies (Jackson Immuno Research Laboratories, Inc., West Grove, PA), followed by enhanced chemiluminescence detection (Biological Industries, BeitHaemek, Israel).

### Real-time PCR

Total RNA was extracted from1 × 10^6^ cells with the Trizol reagent (Bio Basic, Inc.), and cDNA was synthesized using 2 μg of total RNA with RevertAid M-MuLV Reverse Transcriptase (Fermentas International, Inc.). Equal amounts of cDNA were taken for transcript PCR amplification, which was carried out using QuantiTect SYBR Green PCR Kits (Qiagen, Inc.). Glyceraldehyde- 3-phosphate dehydrogenase (GAPDH) was used as an internal standard. The primers used for PCR were as follows: PU.1: forward 5′-ATGTGCCTCCAGTACCCATC-3′, reverse 5′-TCTTCTGGTAGGTCATCTTC-3′; CEBPB: forward 5′-ACAGCGACGAGTACAAGATCC-3′, reverse 5′-GCAGCTGCTTGAACAAGTTCC-3′; CEBPE: forward 5′-CAGCCACTCGAGTTCTCAGG-3′, reverse 5 ′-TGGCTTCACGGCAAAGAGAT-3′; RARB: forward 5′-TTCAGTGCAAGGGAGATCT-3′, reverse 5′-GACGGACTCGCAGTGTAGAAATC-3′; PXN: forward 5′-CATGTACGTCCCCACGAACTG-3′; reverse 5′-CACTGCTGAAATATGAGGAAGAGATG-3′. The PCR protocol consisted of thermal cycling as follows: an initial denaturation at 95 °C for 2 min followed by 40 cycles of 95 °C for 20 s; 58 °C for 30 s; and 72 °C for 30 s using an Eppendorfep Gradient Mastercycler (Eppendorf, Hamburg, Germany). In all of the experiments, two negative controls were carried through all of the steps.

### Virus production and lentiviral transduction

Recombinant lentiviruses were produced by co-transfecting the 293FT cells with pCCL-RARα (a kind gift from Dr. Lingtao Wu at the University of Southern California or a STAT1 shRNA expression plasmid (RHS4533-NM_007315; Open Biosystems), pRΔ8.9 packaging plasmids and pMD.G envelope plasmids. Supernatants containing the infectious virus particles were collected 48 h after the transfection and then used to transduce the AML cells by spinoculation in the presence of polybrene (6 μg/mL). The expression of RARα or STAT1 was analyzed by western blot.

### Statistical analysis

ANOVA or Student unpaired, 2-tailed t tests were used when appropriate.

## Results

### TAK165 inhibits cell proliferation and induces G_0_/G_1_ cell cycle arrest in AML cells

**Figure 1:**
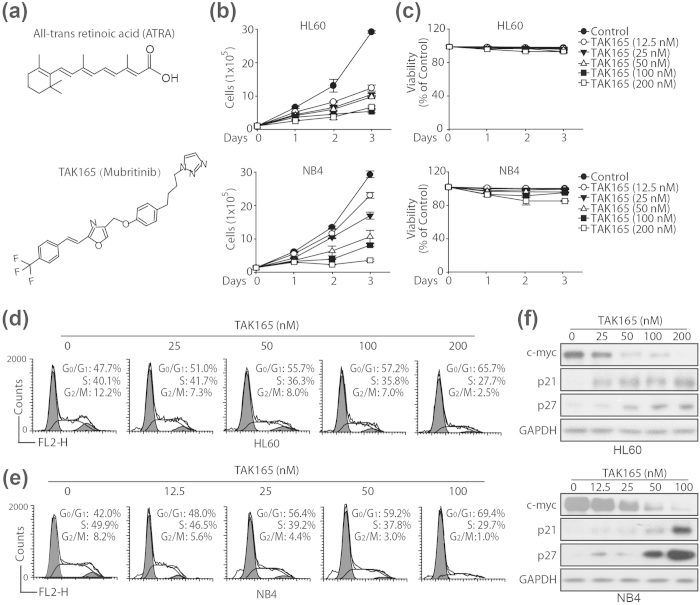
Effect of TAK165 on AML cell proliferation and cycle distribution. (**a**) The chemical structures of TAK165 and ATRA. (**b**,**c**) HL60 and NB4 cell proliferation assay and trypan blue viability assay. The cells were treated with the indicated concentrations of TAK165 for 3 days, and the number of cells was counted each day. The data represent the means ± SD of 3 independent experiments. (**d**,**e**) HL60 and NB4 cell flow cytometric cycle proportion assay. The cells were treated with the indicated concentrations of TAK165 for 3 days. (**f**) A western blot analysis of c-myc, p21 and p27 protein in HL60 and NB4 cells. The cells were treated with the indicated concentrations of TAK165 for 3 days.

First, to determine whether TAK165 triggered the growth arrest of AML cells, both the HL60 and NB4 cells were exposed to serial concentrations of TAK165 for the indicated days. In the HL60 cells, compared to the untreated control group, 12.5 to 200 nM TAK165 significantly inhibited cell proliferation (Fig. 1b, top panel) without any cell death, as evaluated by trypan blue staining (Fig. 1c, top panel). In the NB4 cells, the same treatment significantly inhibited cell proliferation in a dose-dependent manner (Fig. 1b, bottom panel), but for the 200 nM TAK165 treatment, there was evidence of cell death (Fig. 1c, bottom panel). These results revealed that TAK165 treatment inhibited AML cell proliferation without inducing cell death.

To study the progression of the TAK165-induced cell cycle arrest, we chose the concentrations 25–200 nM in the HL60 cells and the concentrations 12.5–100 nM in the NB4 cells for further investigation. As shown in Fig. 1d,e, an increase in the proportion of cells in the G0/G1 phase was observed in the HL60 and NB4 cells when treated with TAK165. In the HL60 cells, compared with 47.7% in the control group, the percentage of G0/G1 cells increased to 65.7% after TAK165 treatment for 3 days (Fig. 1d). Similar results were also obtained in the NB4 cells (Fig. 1e). Consistently, TAK165 significantly decreased the protein expression of c-myc, which is involved in leukemic cell proliferation, and increased the protein expression of p21 and p27, which play important roles in preventing cell cycle progression, in a dose-dependent manner (Fig. 1f). Taken together, these findings indicated that TAK165 inhibits the proliferation of AML cells, which is in parallel with a G0/G1 cell cycle arrest without cytotoxicity.

### TAK165 sensitizes AML cells to ATRA-induced differentiation

**Figure 2:**
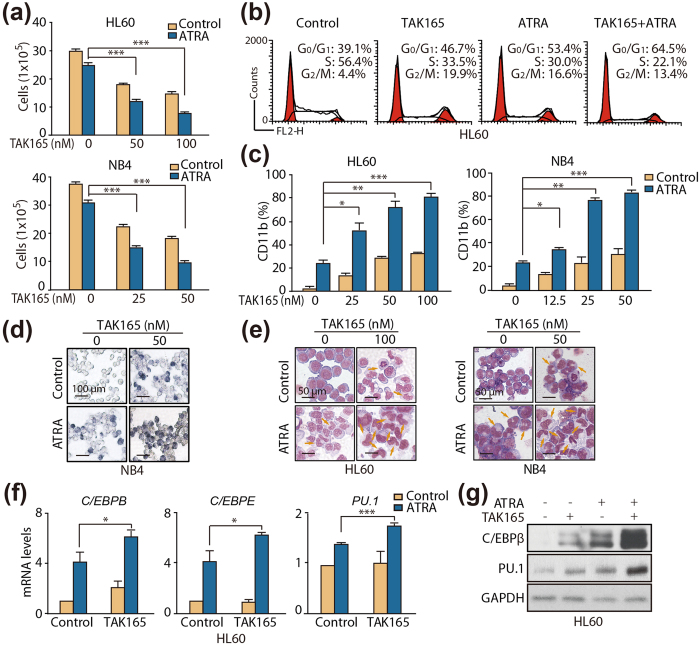
Effect of TAK165 on ATRA-induced AML cell differentiation. (**a**) HL60 and NB4 cell proliferation assay. The cells were treated with the indicated concentrations of TAK165 in the presence of vehicle or ATRA (1 μM for HL60, 2 nM for NB4) for 3 days. (**b**) HL60 cell flow cytometric cycle proportion assay. The cells were treated with 100 nM TAK165 in the presence of vehicle or 1 μM ATRA for 3 days. (**c**) CD11b expression in the HL60 and NB4 cells. The cells were treated with the indicated concentrations of TAK165 in the presence of vehicle or ATRA (1 μM for HL60, 2 nM for NB4) for 3 days. (**d**) NB4 cell NBT-reducing activity. The cells were treated with 50 nM TAK165 in the presence of vehicle or 2 nM ATRA for 3 days. (**e**) HL60 and NB4 cell morphologic differentiation. The cells were treated with the indicated concentrations of TAK165 in the presence of vehicle or ATRA (1 μM for HL60, 2 nM for NB4) for 3 days. (**f**) *CEBPB*, *CEBPE* and *PU.1 *mRNA levels in the HL60 cells as determined by real-time PCR. The cells were treated with 100 nM TAK165 in the presence of 1 μM ATRA for 1 day. GAPDH expression was used as an internal control gene. (**g**) Western blot analysis of PU.1 and C/EBPβ in the HL60 cells. The cells were treated with 100 nM TAK165 in the presence of 1 μM ATRA for 3 day. In (**a**,**c**,**f**), the data are presented as the mean ± SD of 3 independent experiments. *p < 0.05; **p < 0.01; ***p < 0.001.

Because the progress of cancer cell differentiation is tightly coupled to growth arrest in the G0/G1 phase and TAK165 obviously inhibits the proliferation of AML cells following cell cycle arrest, we wondered whether there were synergistic effects between TAK165 and ATRA on AML cell proliferation and differentiation. As expected, TAK165 significantly enhanced the ATRA-induced growth arrest of the HL60 and NB4 cells in a dose-dependent manner (Fig. 2a). Consistently, TAK165 also strengthened the accumulation of AML cells in the G0/G1 phase of the cell cycle induced by ATRA (Fig. 2b and Fig. S1a). Furthermore, to determine whether the TAK165- and ATRA- mediated growth inhibition resulted from myeloid differentiation, we assessed differentiation using standard assays for myeloid maturation. In the HL60 cells, TAK165 significantly promoted ATRA-induced differentiation in a dose-dependent manner, as assessed by CD11b expression (Fig. 2c, left). The fraction of cells expressing CD11b increased from 24.8% ± 3.0% in the 2 nM ATRA-treated group to 80.0% ± 3.4% in the ATRA and TAK165 (100 nM) combination group (*p* < 0.001 vs. the ATRA group). A similar increase in CD11b expression was also observed in the NB4 cells (Fig. 2c, right). To corroborate the differentiating effect of TAK165 and ATRA, the presence of functionally mature myeloid cells was also assessed by NBT reduction assay. Consistent with CD11b expression, a significant enhancement in NBT-positive NB4 cells was observed after TAK165 treatment when compared with the ATRA alone group (Fig. 2d, Fig. S1b).

Moreover, the enhanced differentiation was also confirmed by morphologic analysis using Wright Giemsa staining. Compared with the untreated cells, ATRA or TAK165 alone treated cells showed modestly decreased nucleus to cytoplasmic ratios, whereas the cells treated with a combination of ATRA and TAK165 had a more mature morphology with an increased cytoplasmic to nuclear ratio and obvious nuclear segmentation (Fig. 2e).

We further analyzed the expression changes in the myeloid regulators C/EBPB, C/EBPE and PU.1 in the AML cells. The real-time PCR results showed obvious increases in the *CEBPE*, *CEBPB* and *PU.1* mRNA levels after the ATRA and TAK165 combination treatment (Fig. 2f, Fig. S1c). Similarly, a protein expression analysis also revealed a strong up-regulation of C/EBPβ and PU.1 after the ATRA and TAK165 co-treatment (Fig. 2g, Fig. S1d). Taken together, the CD11b expression, the NBT reduction tests, the morphologic changes, and the myeloid regulator expression changes clearly suggest that the combination of TAK165 and ATRA to induce myeloid differentiation of AML cells has great potential.

### Her2 pathway might not be essential for myeloid differentiation induced by the TAK165 and ATRA combination treatment

**Figure 3:**
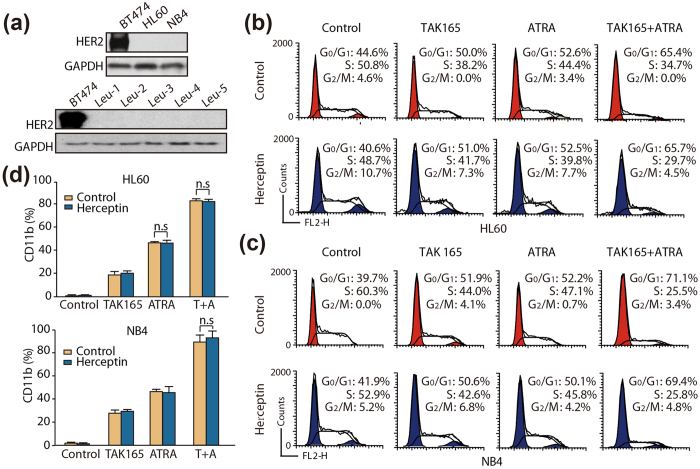
Effect of TAK165 and ATRA on the HER2 pathway. (**a**) Western blot analysis of HER2 in the HL60, NB4 and 5 primary cells from AML patients. (**b**,**c**) HL60 and NB4 cell flow cytometric cycle proportion assay. The cells were treated with TAK165 (100 nM for HL60, 50 nM for NB4) and ATRA (1 μM for HL60, 2 nM for NB4) in the presence of vehicle or Herceptin (400 μg/ml) for 3 days. (**d**) CD11b expression in the HL60 and NB4 cells. The cells were treated with TAK165 (100 nM for HL60, 50 nM for NB4) and ATRA (1 μM for HL60, 2 nM for NB4) in the presence of vehicle or Herceptin (400 μg/ml) for 3 days. The data represent the mean ± SD of 3 independent experiments. n.s, *p* > 0.05.

To gain insight into the mechanisms underlying the synergy between TAK165 and ATRA, we first examined whether TAK165-mediated HER2 inhibition contributed to the combination treatment-induced differentiation, given that TAK165 is a specific HER2 inhibitor. First, we attempted to determine the expression levels of the HER2 protein in the two AML cell lines. Unexpectedly, the HER2 protein was undetectable in the HL60 and NB4 cells, but a strong HER2 expression was found in the positive control group, a breast cancer cell line, BT474 (Fig. 3a). Furthermore, we also detected Her2 expression in 5 primary AML cells. As illustrated in Fig. 3a, Her2 was also undetectable in all those primary cells from AML patients when combined with positive control (BT474 cells).

Herceptin (Trastuzumab), an anti-HER2/neu receptor monoclonal antibody^12^,^13^, was further used to evaluate the role of HER2 during the ATRA-induced differentiation of AML cells. As shown in Fig. 3b,c, Herceptin did not enhance ATRA-induced cell cycle arrest, while TAK165 did. Meanwhile, Herceptin also had no effect on the ATRA and TAK165 combination treatment induced cell cycle arrest. In addition, there were no obvious alterations in the myeloid differentiation driven by either ATRA alone or ATRA/TAK165 combination as assessed by CD11b expression (Fig. 3d). Thus, our data suggested that HER2 pathway might not be essential for myeloid differentiation induced by the TAK165 and ATRA combination treatment.

### RARα activation is critical for the differentiation induced by the TAK165 and ATRA combination treatment

**Figure 4:**
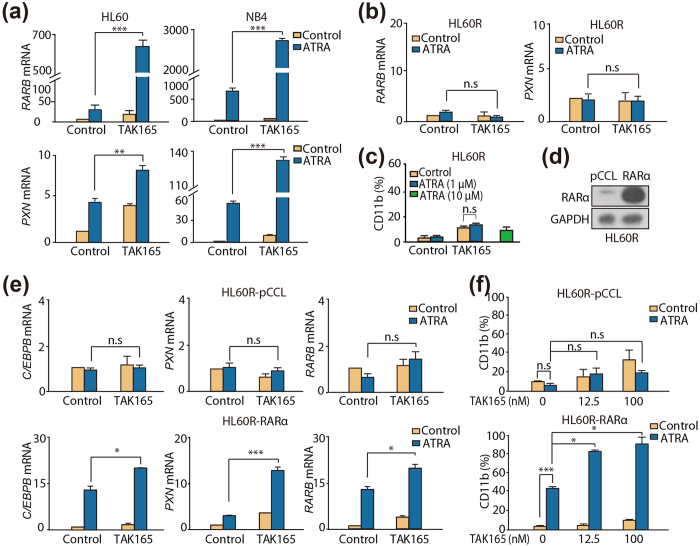
Effect of TAK165 and ATRA on RARα activation. (**a**,**b**) *RARB* and *PXN* mRNA levels in HL60, NB4 and HL60R cells as determined by real-time PCR. The cells were treated with TAK165 (100 nM for HL60 and HL60R, 50 nM for NB4) and ATRA (1 μM for HL60 and HL60R, 2 nM for NB4) for 1 day. GAPDH expression was used as an internal control gene. (**c**) CD11b expression in the HL60R cells. The cells were treated withTAK165 (100 nM) in the presence of vehicle or 1 μM ATRA for 3 day. A 10 μM ATRA treatment was used to indicate the resistance of the HL60R cells to ATRA. (**d**) Western blot analysis of RARα in the HL60R cells transduced with the different lentivirus. (**e**) *CEBPB*, *RARB* and *PXN* mRNA levels in the HL60R-pCCL and HL60R-RARα cells as determined by real-time PCR. The cells were treated with TAK165 (100 nM) in the presence of vehicle or 1 μM ATRA for 1 day. (**f**) CD11b expression in the HL60R-pCCL and HL60R-RARα cells. The cells were treated withTAK165in the presence of vehicle or 1 μM ATRA for 3 days. In (**a**,**b**,**e**,**f**), data presented are the mean ± SD of 3 independent experiments. *p < 0.05; **p < 0.01; ***p < 0.001.n.s, *p* > 0.05.

Because HER2 is not the target of TAK165 and ATRA and the transcriptional activation of RARα is generally considered to be the classical mechanism for the differentiating effect of ATRA^14^,^15^,^16^,^17^, we then hypothesized that TAK165 might potentiate the RARα sensitization of ATRA on AML cells. As expected, increased expression of the RARα target genes *RARB* and *PXN* were observed in the ATRA single treatment group. TAK165 further enhanced the ATRA-triggered transcription of *RARB* and *PXN* (Fig. 4a), indicating that the activation of RARα is involved in the TAK165 and ATRA induced differentiation of AML cells.

Next, AML HL60R cells, harboring a RARαΔAF-2 domain and exhibiting relative resistance to RA, were used to determine the differentiation induction effect of ATRA and TAK165^18^. As illustrated in Fig. 4b, in sharp contrast to the ATRA-responsive cells (HL60 and NB4, Fig. 4a), the HL60R cells showed no changes in the mRNA expression of *RARB* and *PXN* when exposed to ATRA or ATRA plus TAK165. Moreover, no visible differentiation, as reflected by CD11b expression, was observed in the HL60R cells treated with ATRA, ATRA plus TAK165 or even a high concentration of ATRA (10 μM) (Fig. 4c). These results suggested that RARα might play an important role in the combination treatment. To further support the concept that RARα is critical for TAK165-enhanced differentiation, we tested whether exogenously expressed wild type RARα in HL60R cells could restore the sensitivity of the HL60R cells to the ATRA-induced differentiation. As shown in Fig. 4d, the HL60R cells were transduced with the lentiviral-vector pCCL or lentiviral-pCCL-RARα. These two HL60R cells were treated with ATRA or TAK165 and then differentiation was determined. Compared with the HL60R-pCCL cells, a significant up-regulation of the RARα target gene mRNA expression (Fig. 4e) and a strong increase in CD11b-positive cells (Fig. 4f) were both detected in the HL60R-RARα cells treated with ATRA or ATRA plus TAK165. These findings indicated that the activation of RARα is critical for AML cells differentiation induced by the treatment of a combination of TAK165 and ATRA.

### TAK165 and ATRA-induced RARα-related STAT1 activation is required for differentiation

**Figure 5:**
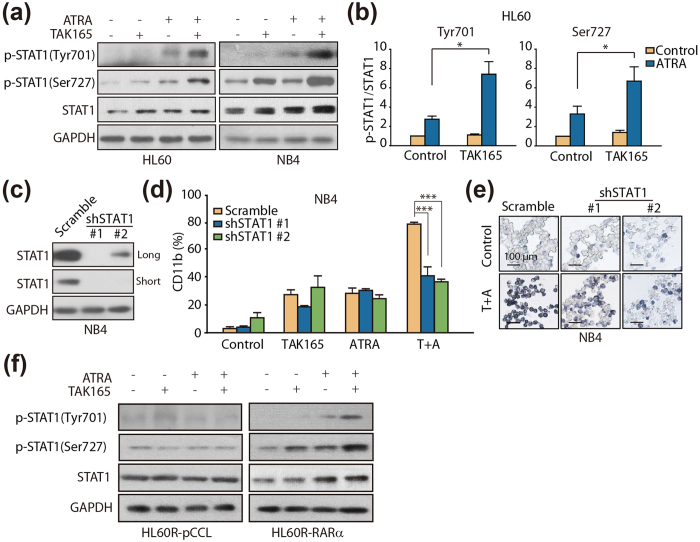
Effect of TAK165 and ATRA on STAT1 activation. (**a**) Western blot analysis of p-STAT1 (Tyr701 and Ser727) and STAT1 in the HL60 and NB4 cells. The cells were treated with TAK165 (100 nM for HL60, 50 nM for NB4) and ATRA (1 μM for HL60, 2 nM for NB4) for 3 days. (**b**) The relative phosphorylation level of STAT1 at Tyr701 and Ser727 in the HL60 cells, were evaluated by p-STAT1 (Tyr701 or Ser727)/STAT1. (**c**) STAT1 expression in the NB4-scrambleand NB4-shRNA-STAT1 cells was measured by western blot. (**d**) CD11b expression in the NB4-scrambleand NB4-shRNA-STAT1 cells. The cells were treated with 50 nM TAK165 and 2 nM ATRA for 3 days. The data presented are the mean ± SD. ***p < 0.001. (**e**) NBT-reducing activity in the NB4-scrambleand NB4-shRNA-STAT1 cells. The cells were treated withTAK165 (50 nM) and ATRA (2 nM) for 3 days. (**f**) p-STAT1 (Tyr701 and Ser727) and STAT1 expression in the HL60R-pCCL and HL60R-RARα cells. The cells were treated withTAK165 in the presence of vehicle or 1 μM ATRA for 3 days.

Numerous reports demonstrate the essential role of STAT1 protein in ATRA-induced myeloid differentiation^19^; hence, we intended to determine whether STAT1 activation was also involved in TAK165 plus ATRA-induced AML cell differentiation. It is reported that phosphorylation on Tyr701 is mandatory for STAT1 dimerization^20^, nuclear translocation and DNA binding, and full transcriptional activity of the homodimer is manifested when Ser727 in the transcription activation domain (TAD) is also phosphorylated^21^. As shown in Fig. 5a,b and Fig. S2b, we observed that significant increase in the phosphorylation of STAT1 (Tyr701 and Ser727) were observed in the TAK165 and ATRA combination treatment group compared with the ATRA or TAK165 single treatment groups, indicating that STAT1 was activated upon the combination treatment-induced differentiation of AML cells. To further evaluate the role of STAT1 in the AML differentiation induced by the TAK165 plus ATRA, we knocked down STAT1 expression with two shRNAs. As shown in Fig. 5c, the expression of shSTAT1 #1 and #2 differently reduced STAT1 protein expression in the NB4 cells compared with the scramble controls. As expected, the silencing of STAT1 expression significantly abolished the ability of TAK165 to sensitize the cells to ATRA. In detail, the STAT1-knockdown cells showed markedly reduced CD11b expression after ATRA plus TAK165 treatment compared with the control cells (80.1% ± 1.21%, 42.0% ± 6.11%, and 37.6% ± 1.57% for scramble, shSTAT1 #1 and shSTAT1 #2, respectively) (Fig. 5d). However, STAT1 knockdown did not obviously affect the differentiation mediated by ATRA alone (Fig. 5d), which may be due to the weak differentiation induced by the low concentration of ATRA. Similar results were also obtained in the NBT reduction assay (Fig. 5e and Fig. S2c). Hence, these results clearly indicated that STAT1 induction contributes to myeloid differentiation upon TAK165 and ATRA treatment.

A RARE domain reportedly exists in the STAT1 promoter^22^. Therefore, the HL60R-pCCL and HL60R-RARα cells were treated with ATRA and TAK165 and STAT1 activity was determined. As shown in Fig. 5f, compared with the HL60R-pCCL cells, significant increase in the phosphorylation of STAT1 (Tyr701 and Ser727) were detected in the HL60R-RARα cells treated with ATRA plus TAK165. These data demonstrated that STAT1 activation induced by TAK165 and ATRA treatment is RARα-related.

### Activation of the RARα/STAT1 axis is dependent on the phosphorylation of the MEK/ERK cascade

**Figure 6:**
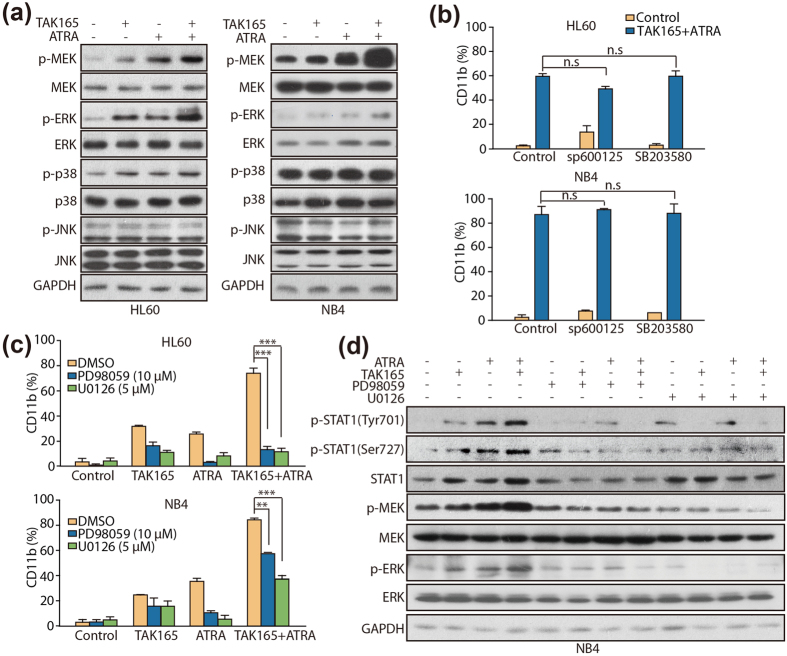
Effect of TAK165 and ATRA on the MEK pathway. (**a**) Western blot analysis of MAPK signaling in the HL60 and NB4 cells. The cells were treated withTAK165 (100 nM for HL60, 50 nM for NB4) and ATRA (1 μM for HL60, 2 nM for NB4) for 3 days. (**b**,**c**) CD11b expression in the HL60 and NB4 cells. The cells were treated with TAK165 (100 nM for HL60, 50 nM for NB4) and ATRA (1 μM for HL60, 2 nM for NB4) in the presence of vehicle or MAPK inhibitors (20 μM SB203580 (p38 inhibitor), 20 μM sp600125 (JNK inhibitor), 10 μM PD98059 (MEK inhibitor), 5 μM U0126 (MEK inhibitor) for 3 days. The data presented are the mean ± SD. ***p < 0.001.n.s, *p* > 0.05. (**d**) Western blot analysis of p-STAT1 (Tyr701 and Ser727), STAT1 and the MEK signal pathway in the NB4 cells. The cells were treated with 50 nM TAK165 and 2 nM ATRA in the presence of vehicle or MAPK inhibitors (10 μM PD98059 or 5 μM U0126) for 3 days.

Given that STAT1 is phosphorylated by MAPK cascades, including MEK, ERKs, p38 and JNKs^23^,^24^, we also detected the effect of ATRA and TAK165 on MAPK. As shown in Fig. 6a, treatment with TAK165 or ATRA alone led to an increase in the phosphorylation of MEK and ERK, and the combination treatment had an even greater effect than the mono-treatments. In contrast, p-p38 and p-JNK were not further activated in the ATRA plus TAK165 co-treated AML cells.

To further determine whether the phosphorylation of the MEK/ERK cascade other than p38 or JNK was indispensable for the induction of AML differentiation by the ATRA and TAK165 combination treatment, the HL60 and NB4 cells were treated with TAK165 and ATRA in the presence of different MAPK inhibitors, and then, the expression of CD11b was analyzed. Little change in the expression of CD11b was detected in all of the cells treated with the JNK inhibitor (sp600125) or the p38 inhibitor (SB203580) (Fig. 6b). However, the MEK/ERK inhibitors (PD98059 and U0126) significantly inhibited the combination treatment-induced CD11b expression in the HL60 and NB4 cells (Fig. 6c). These results further indicated that the phosphorylation of the MEK/ERK cascade contributed to the differentiation induced by ATRA and TAK165. As expected, the activation of MEK and ERK was successfully inhibited by PD98059 and U0126 (Fig. 6d). In addition, the phosphorylation of STAT1 (Tyr701 and Ser727) was also strikingly inhibited by PD98059 and U0126 in the AML cells, indicating that the TAK165 and ATRA combination treatment-induced activation of STAT1 was dependent on the activation of the MEK/ERK-cascade. In summary, our data suggested that TAK165 promotes ATRA-induced differentiation by activating the MEK/ERK-mediated RARα/STAT1 axis.

## Discussion

ATRA is an effective inducer of APL cell differentiation and markedly improves the survival and prognosis of patients with this disease^25^. The success of ATRA in APL highly encourages researchers to apply it in other types of AML. However, AML, with the exception of APL, fails to respond to pharmacologic doses of ATRA^26^,^27^. In our study, synergistic differentiation effects were achieved by a TAK165 and ATRA combination treatment in AML cells. Of note, we found that the biological function of the combination of TAK165 and ATRA in inducing cell differentiation is driven by the activation of the RARα/STAT1 axis resulting from MEK/ERK phosphorylation instead of HER2 inhibition.

Because TAK165 is a specific HER2 inhibitor, which is also reported to decrease cell proliferation and inhibit the G1/S transition in leukemia cells^28^, we intended to evaluate whether TAK165 enhances the effect of ATRA by inhibiting HER2. Contrary to expectations, although TAK165 and ATRA had a synergistic effect on AML cell differentiation, HER2 might not be essential for the combination treatment. HER2 was weakly expressed in AML cells, and no marked changes could be captured in AML cell proliferation and differentiation in the presence of another HER2 inhibitor (Herceptin) (Fig. 3). Consistent with our findings, previous studies also observed that TAK165 significantly inhibited tumor growth compared with Herceptin in urological cancers with weak HER2 expression^9^, and TAK165-inhibted cell proliferation might be regulated by the suppression of ERK phosphorylation instead of HER2 inhibition^29^. Thus, it is possible that HER2 inhibition is not necessary for TAK165 to enhance ATRA-induced differentiation. Therefore, a structural modification of TAK165 that maintains its synergistic ability in differentiation but does not target HER2 should be performed to better understand the specific mechanisms of TAK165 in enhancing the differentiation induced by ATRA.

RARα plays a critical and central role in mediating the RA-induced terminal differentiation of leukemia cells. We first determined whether RARα was activated in TAK165 and ATRA-induced differentiation. A significant increase in the expression of the RARα target genes was observed in the HL60 and NB4 cells after TAK165 and ATRA treatment, while the changes were lost in the HL60R cells, which harbor a mutated RARα gene with little differentiation effects. However, when the RARα target genes were up-regulated as a result of RARα over-expression in the HL60R cells, the differentiation capacity of TAK165 and ATRA was recovered. These results demonstrated that RARα is physiologically implicated in the regulation of TAK165 and ATRA-mediated differentiation. Moreover, as shown in Fig. 5f, compared with the HL60R-pCCL cells, a significant activation of STAT1 was detected in the HL60R-RARα cells treated with ATRA plus TAK165, which further indicated that the differentiation induced by ATRA and TAK165 combination is RARα-dependent.

STAT family proteins, activated by cytokines, hormones, and growth factors function as transcription factors that mediate a variety of biologic processes, such as cell proliferation and apoptosis^30^. STAT1 plays a key role in the ATRA-induced terminal differentiation of myeloid cells, through regulation of cell-cycle proteins and myeloid-specific transcription factors^31^,^32^,^33^,^34^. Here, phosphorylation of STAT1 was observed in TAK165 and ATRA induced differentiation, accompanied by cell cycle arrest. Then, the knockdown of STAT1 by short hairpin RNA (shRNA) substantially decreased CD11b expression and the NBT-positive cell level after the combination therapy. These results all support the notion that STAT1 activation may contribute to TAK165 and ATRA induced myeloid differentiation.

In addition, the protein level of STAT1 slightly increased upon ATRA single treatment. However, when compared to the ATRA or TAK165 single treatment, the protein level of STAT1 in ATRA and TAK165 combination group was only up-regulated in NB4 cells, not in HL60 cells. We also semi-quantitated the western blot results of STAT1, similar changes were observed (Fig. S2a), suggesting that the STAT1 up-regulation was not universal phenomena after ATRA and TAK165 combination treatment in AML cells. Moreover, the western blot results of p-STAT1 (Tyr701), p-STAT1 (Ser727) and STAT1 were semi-quantitated, and the relative p-STAT1 (Tyr701) and p-STAT1 (Ser727) were normalized with STAT1. As shown in Fig. 5b and Fig. S2b, we found that the relative phosphortlation of STAT1 (Tyr701 and Ser727) increased significantly in ATRA and TAK165 combination group. Therefore, out results highly suggested that STAT1 activation is mainly resulted from phosphorylation at Tyr701 and Ser727 other than the expression regulation.

Previous studies show that STAT1 is phosphorylated by MAPK cascades, including MEK, ERK, p38 and JNK^19^. MEK/ERK signaling is widely reported to play a vital role in the differentiation of various types of cells, including hematopoietic cells^35^,^36^. Data obtained from the western blot revealed that MEK and ERK are strongly activated in TAK165 and ATRA treated AML cells, while p38 and JNK are not. Importantly, the differentiation effects induced by the combination treatment were mostly inhibited by the MEK/ERK inhibitors (PD98059 and U0126), with the exception of the p38 or JNK inhibitor. Moreover, the phosphorylation of STAT1 also decreased when MEK/ERK activity was depressed by the inhibitors, further proving that MEK/ERK signaling is a key intermediate in the STAT1 sensitization mediated by TAK165 and ATRA. MAPK, including MEK/ERK, is essential for the phosphorylation of the nuclear RA receptor family members, which appear to influence the ability of ATRA to induce receptor-dependent transcriptional activation, cell growth arrest, and differentiation^37^,^38^,^39^. However, the precise relationship between RARα activation and MEK/ERK-dependent STAT1 activation requires further study.

In conclusion, we present evidence showing enhanced ATRA therapeutic activity upon combination with TAK165. Our study also shows that the TAK165 and ATRA combination treatment synergistically induces differentiation by activating the MEK/ERK-mediated RARα/STAT1 axis. Although further clinical and mechanistic studies are needed for this combination treatment, the data presented here warrant that ATRA coupled with TAK165 may lead to new applications of differentiation-based approaches for AML and other leukemia therapies.

## Additional Information

**How to cite this article**: Shao, X. *et al*. The HER2 inhibitor TAK165 Sensitizes Human Acute Myeloid Leukemia Cells to Retinoic Acid-Induced Myeloid Differentiation by activating MEK/ERK mediated RARα/STAT1 axis. *Sci. Rep*. **6**, 24589; doi: 10.1038/srep24589 (2016).

## Supplementary Material

Supplementary Information
