# Supplementary material for: The HER2 inhibitor TAK165 Sensitizes Human Acute Myeloid Leukemia Cells to Retinoic Acid-Induced Myeloid Differentiation by activating MEK/ERK mediated RARα/STAT1 axis
Source: Sci Rep. 2016 Apr 14;6:24589. doi: 10.1038/srep24589 (PMC4830980; doi:10.1038/srep24589)
Supplement: Supplementary Information [file srep24589-s1.pdf]

The HER2 inhibitor TAK165 Sensitizes Human Acute Myeloid Leukemia Cells to Retinoic Acid-Induced Myeloid Differentiation by activating MEK/ERK mediated RARα/STAT1 axis

Xuejing Shao[#], Yujia Liu[#], Yangling Li, Miao Xian, Qian Zhou, Bo Yang, Meidan Ying* and Qiaojun He*

Zhejiang Province Key Laboratory of Anti-Cancer Drug Research, Institute of Pharmacology and Toxicology, School of Pharmaceutical Sciences, Zhejiang University, Hangzhou, Zhejiang, China.

*Corresponding Authors: Meidan Ying, Ph.D. and Qiaojun He, Ph.D., Room 113, Institute of Pharmacology and Toxicology, College of Pharmaceutical Sciences, Zhejiang University, Hangzhou, China,310058.Phone/Fax: 86-571-88208400; E-mail: Mying@zju.edu.cn (M. Ying); qiaojunhe@zju.edu.cn (Q. He).

# These authors contribute equally to this manuscript.

Supplementary Figures

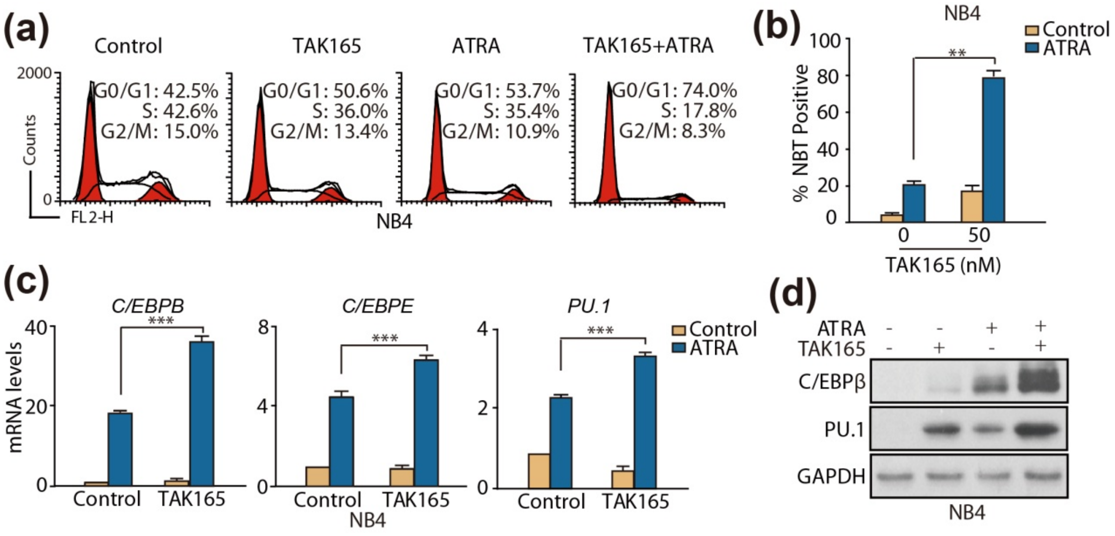

Supplementary Fig. S1. Effect of TAK165 and ATRA combination on NB4 cell Differentiation

(a) NB4 cell flow cytometric cycle proportion assay.Cells were treated with 50nM TAK165 in the presence of vehicle or 2 nM ATRAfor 3 days. (b) NBT-positive NB4 cells were counted in the presence of TAK165 and ATRA (2nM) for 3 days. (c)*CEBPB*, *CEBPE*and*PU.1* mRNA levels in NB4 cell as determined by real-time PCR. Cells were treated with 50nM TAK165 in the presence of 2nM ATRA for 1 day. GAPDH expression was used as an internal control gene. (d) Western blot analysis of PU.1, C/EBPβ in NB4. Cells were treated with 50nM TAK165 in the presence of 2nM ATRA for 3 day. (a)-(c), the data are presented as the mean ± SD of 3 independent experiments. **, p<0.01; ***, p<0.001.

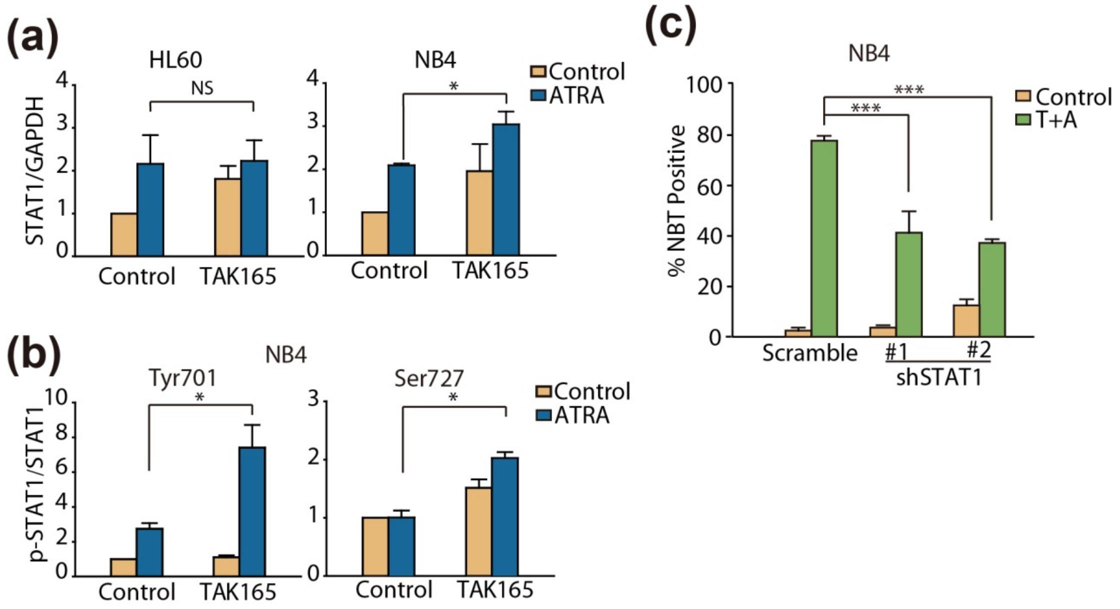

Supplementary Fig. S2. Effect of STAT1 activation on TAK165 and ATRA combination in NB4 cell differentiation

(a) The relative STAT1 level of AML cells was detected by STAT1/GAPDH. (b) The relative phosphorylation level of STAT1 at Tyr701 and Ser727 in the NB4 cells, were evaluated by p-STAT1 (Tyr701 or Ser727) / STAT1. (c) CD11b-positive cells were counted in NB4-scrambleand NB4-shRNA-STAT1 cells. NB4 cell was treated with TAK165 (50nM) and ATRA (2nM) for 3 days.***, $p<0.001$. The data represent the mean ± SD of 3 independent experiments.
